# Prognostic role of pre-sarcopenia and body composition with long-term outcomes in obstructive colorectal cancer: a retrospective cohort study

**DOI:** 10.1186/s12957-020-02006-3

**Published:** 2020-08-28

**Authors:** Chul Seung Lee, Daeyoun David Won, Soon Nam Oh, Yoon Suk Lee, In Kyu Lee, In-Ho Kim, Moon Hyung Choi, Seong Taek Oh

**Affiliations:** 1 Department of Surgery, Seoul St. Mary’s Hospital, College of Medicine, The Catholic University of Korea, Seoul, Republic of Korea; 2 Graduate School of Medicine, The Catholic University of Korea, Seoul, Republic of Korea; 3 Department of Radiology, Seoul St. Mary’s Hospital, College of Medicine, The Catholic University of Korea, Seoul, Republic of Korea; 4 Cancer Research Institute, College of Medicine, The Catholic University of Korea, Seoul, Republic of Korea; 5 Division of Medical Oncology, Department of Internal Medicine, Seoul St. Mary’s Hospital, College of Medicine, The Catholic University of Korea, Seoul, Republic of Korea; 6 Department of Radiology, Eunpyeong St. Mary’s Hospital, College of Medicine, The Catholic University of Korea, Seoul, Republic of Korea; 7 Department of Surgery, Uijeongbu St. Mary’s Hospital, College of Medicine, The Catholic University of Korea, 271, Cheonbo-Ro, Uijeongbu-si, Gyeonggi-do, 11765 Seoul, Republic of Korea

**Keywords:** Sarcopenia, Body composition, Colorectal neoplasms, Self-expandable metallic stents

## Abstract

**Background:**

The clinical significance of pre-sarcopenia in colorectal cancer obstruction has not yet been described. The present study aimed to determine the short- and long-term oncologic impacts of pre-sarcopenia in obstructive colorectal cancer.

**Methods:**

We retrospectively analyzed 214 patients with obstructive colon cancer between January 2004 and December 2013. Initial staging computed tomography (CT) scans identified pre-sarcopenia and visceral obesity by measuring the muscle and visceral fat areas at the third lumbar vertebra level. Both short-term postoperative and long-term oncologic outcomes were analyzed.

**Results:**

Among all 214 patients, 71 (33.2%) were diagnosed with pre-sarcopenia. Pre-sarcopenia had a negative oncologic impact in both disease-free survival (DFS) and overall survival (OS), (hazard ratio [HR] = 1.86, 95% confidence interval [CI] 1.04–3.13, *p* = 0.037, and HR = 1.92, CI 1.02–3.60, *p* = 0.043, respectively). Visceral adiposity, body mass index (BMI), and neutrophil-lymphocyte ratio (NLR) did not significantly impact DFS and OS.

**Conclusion:**

Pre-sarcopenia is a clinical factor significantly associated with OS and DFS but not with short-term complications in obstructive colorectal cancer. In future, prospective studies should incorporate body composition data in patient risk assessments and oncologic prediction tools.

## Background

Sarcopenia is defined as a decrease in skeletal muscle volume and function [1] that has been reported to reflect patients’ frailty. Major clinical guidelines have incorporated sarcopenia as a tool to assess cachexia in cancer patients [2]. Sarcopenia patients may possess unfavorable nutritional and immunological factors [3] and show lower compliance to consecutive anti-tumor treatments such as radiotherapy, surgery, and chemotherapy [4].

Sarcopenia, especially in obese patients, is not easily characterized by overall weight loss or decreased body mass index (BMI) alone [5]. With an increased prevalence of obesity, patients with both sarcopenia and obesity (sarcopenic obesity) are at a higher risk of adverse outcomes [6] and mortality [7]. Thus, measuring the skeletal muscle mass and quality with BMI alone may not be appropriate in such cases. As reported, not only sarcopenia, but visceral obesity has also shown increased incidence of post-operative complication and delayed recovery [8, 9]. Further, preoperative computerized tomography (CT) scan by assessing the muscle area in the third lumbar vertebra (L3) region [10] appears to be one of the most widely researched topics in retrospective studies to measure skeletal muscle index (SMI) and to define pre-sarcopenia. Pre-sarcopenia is characterized by low muscle mass without impact on muscle strength [11].

Pre-sarcopenia is a significant prognostic factor in colorectal cancer patients. In colorectal cancer surgery, pre-sarcopenia has been reported to predict poorer postoperative short-term and oncologic long-term outcomes [12, 13]. Pre-sarcopenia patients undergoing resection of colorectal liver metastases have a shorter median survival and lower disease-free survival (DFS) rates than non-pre-sarcopenia patients [14]. Additionally, pre-sarcopenia was negatively associated with overall survival (OS) in locally advanced rectal cancer patients who underwent neoadjuvant chemoradiation therapy and curative resection [15]. Despite this, the clinical importance of pre-sarcopenia in obstructive colorectal cancer has not been fully described.

Obstructive colon cancer is a significant clinical event that affects the short- and long-term prognosis of patients. The rates of mortality and complications associated with emergency surgery for obstructive colorectal cancer are higher than those associated with elective surgeries [16, 17]. Regarding long-term oncologic prognosis, colon cancer obstruction has a negative impact on DFS and OS [18, 19]. Thus, treatment guidelines recommend adjuvant chemotherapy for this high-risk group [20].

Intestinal decompression using a self-expandable metallic colonic stent (SEMS) allows bowel preparation, medical stabilization, and optimization of comorbid illnesses, theoretically improving the patient’s nutritional and inflammatory status. The Controlling Nutritional Status (CONUT) Score—a cumulative score calculated from the serum albumin level, total cholesterol level, and total lymphocyte count—is used as a nutritional screening tool. This score is easily calculated and found to be significantly associated with the Subjective Global Assessment (SGA) and Full Nutritional Assessment (FNA) tests [21]. Recent studies show that the preoperative CONUT score is an independent prognostic factor for cancer-specific survival and disease-free survival (DFS) in obstructive colorectal cancer patients [22].

Neutrophils play roles in both inflammation and cancer and have been implicated as key drivers of oncogenesis associated with postoperative inflammation [23, 24]. The neutrophil-to-lymphocyte ratio (NLR) has, hence, been proposed as an indicator of postsurgical inflammation-mediated tumor stimulation. NLR has also been reported to be an independent prognostic factor after resection of colorectal cancer [25–27].

Few studies have described the prognostic role of pre-sarcopenia diagnosed by L3SMI in obstructive colorectal cancer, as compared to nutritional or inflammatory factors. The present study aims to determine if the short-term and long-term oncological effects of pre-sarcopenia, diagnosed by L3SMI, may play a decisive role when compared to other factors in obstructive colorectal cancer.

## Materials and methods

### Definitions

We have defined obstructive colorectal cancer as pathological confirmation of adenocarcinoma originated from the cecum to rectum with clinical symptom of obstruction (abdominal distention, pain, tenderness, and no stool passage) and radiological finding of obstruction in computed tomography (CT) scan or failure of scope passing beyond cancer lesion. Also, we included only complete obstruction to this definition, which clinically required immediate procedure. The surgeon finally decided whether patients had a complete obstruction or not. OS was defined as the period of time between the surgery and death of any cause. DFS was defined as the period of time between the surgery and disease of any cause. OS was calculated from date of surgery until death of any cause or censored at last follow-up.

### Subjects

Between January 2004 and December 2013, a total of 214 consecutive patients with obstructive colorectal cancer were enrolled. The inclusion criteria were as follows: (1) Clinically (abdominal distention, pain, tenderness, and no stool passage) and radiologically confirmed malignant large bowel obstruction and (2) the patients who required immediate procedure due to complete obstruction. Colon perforation, metastatic cancer, and nonradical resection have been reported to be poor prognostic factors [28–30]. The exclusion criteria were as follows: (1) palliative bypass surgery, (2) palliative stoma surgery, (3) palliative bypass surgery, (4) metastatic cancer, and (5) colon perforation. During initial staging computed tomography (CT), body composition evaluations were performed at the level of the transverse process of third lumbar vertebra as previously described [15]. The total body fat area, visceral fat area, subcutaneous fat area, and abdominal circumference were measured automatically (TeraRecon Aquarius Workstation, TeraRecon, Foster City, California, USA), and visceral fat area was used to identify visceral adiposity. The skeletal muscle areas (psoas, para-spinal, transverse abdominis, rectus abdominis, internal oblique, and external oblique muscles) were measured using a commercial system (Advantage Windows workstation, GE Healthcare, Milwaukee, Wisconsin, USA) entailing Hounsfield unit thresholds between − 29 to + 150 [31]. To normalize the skeletal muscle area for patient height, skeletal muscle index (SMI) was calculated as the skeletal area (cm^2^) divided by the square of the height (m^2^). Pre-sarcopenia was defined using sex-specific cutoff values for SMI (46.4 cm^2^ /m^2^ for men and 37.5 cm^2^/m^2^ for women) [32]. The cutoff CT visceral fat area for the classification of visceral obesity has not yet been standardized. Previous studies have used 100 cm^2^ and 130 cm^2^ as cutoffs [33]. The present study defined visceral obesity as an area of visceral fat of 130 cm^2^ and above. Left colon cancer was defined as cancer arising between the distal third of the transverse colon and the rectosigmoid junction of the colon. The CONUT score was calculated using the peripheral lymphocyte count, serum albumin, and total cholesterol concentrations. (1) Albumin concentrations of ≥ 3.5, 3.0–3.49, 2.5–2.99, and < 2.5 g/dL were scored as 0, 2, 4, and 6 points, respectively; (2) total lymphocyte counts of ≥ 1600, 1200–1599, 800–1199, and < 800/mm^3^ were scored as 0, 1, 2, and 3 points, respectively; and (3) total cholesterol concentrations of ≥ 180, 140–179, 100–139, and < 100 mg/dL were scored as 0, 1, 2, and 3 points, respectively. Accordingly, the sum of (1), (2), and (3) was the final CONUT score.

Emergency surgery or self-expandable metallic stent (SEMS) placement was performed based on previously published data [34]. The surgeon determined the operative method for each case considering the patient’s medical status and tumor location. A SEMS (HANARO stent, M.I. Tech Co., Ltd, Seoul, South Korea, or Niti-S stent, Taewoong Medical, Co., Ltd, Gyeonggido, South Korea) was inserted through the working channel over the guidewire under fluoroscopic guidance.

This study was approved by the St. Mary’s Hospital Research Ethics Board (KC19RESI0152), and informed consent was obtained from all participants.

### Statistical analysis

Chi-square and Fisher’s exact tests were performed for association analysis. *P* values < 0.05 were considered statistically significant. Differences between groups were evaluated using Student’s *t* and *x*^2^ tests for continuous and categorical variables, respectively. Categorical variables were reported as numbers and percentages, and distribution of continuous characteristics was reported as median (interquartile range [IQR]) or mean ± standard deviation (SD). OS and DFS curves were analyzed using the Kaplan–Meier method and compared by log-rank test for univariate analysis. Hazard ratios (HRs) were estimated with a Cox proportional hazards regression model. A multivariable Cox regression analysis was carried out to examine whether the different variables were associated with disease recurrences and overall survival (OS). For backward conditional Cox proportional hazards analysis, variables were chosen according to *p* < 0.05 in univariate analysis. Statistical analyses were performed using SPSS version 24.0 (IBM SPSS Statistics®, Armonk, NY, USA). All of the tests were 2 sided, and *p* < 0.05 was considered significant.

## Results

A total of 214 patients (126 men and 90 women) were enrolled in this study. The detailed demographics and clinical treatment courses according to pre-sarcopenia status are summarized in Table 1. Seventy-one patients had pre-sarcopenia based on initial CT. Tumor-related factors (tumor location, carcinoembryonic antigen [CEA]), pathologic factors (stage, lymph node status, lymph-vascular invasion), patient-related factors (American Society of Anesthesiologist [ASA] physical status), and nutritional factor (CONUT score) did not differ significantly according to pre-sarcopenia status (Table 1). Pre-sarcopenia was observed more frequently in male patients than in female patients (51 men, 70.8% vs 75 men, 52.1%, *p* = 0.008). Table 2 shows the association between pre-sarcopenia and body composition factors. The mean BMIs in the non-pre-sarcopenia and pre-sarcopenia groups did not show significant difference based on sex (*p* = 0.486 and 0.687, respectively).

**Table 1:** Clinicopathologic characteristics according to pre-sarcopenia status

	Non-pre-sarcopenia (*N* = 143)	Pre-sarcopenia (*N* = 71)	*P*
Sex, male (%)	75 (52.1)	51 (70.8)	0.008
Age (years), > 70 (%)	29 (20.1)	21 (29.2)	0.138
Location (%)			0.958
Right	32 (22.2)	16 (22.2)	
Left	94 (65.3)	46 (63.9)	
Rectum	18 (12.5)	10 (13.9)	
Preoperative CEA, ≥ 5 (%)	55 (40.4)	26 (40.0)	0.952
Neutrophil lymphocyte ratio, ≥ 5 (%)	54 (76.1)	17 (23.9)	0.932
ASA (%)			0.466
I	42 (29.2)	18 (25.0)	
II	91 (63.2)	46 (63.9)	
III	11 (7.6)	7 (9.7)	
IV	0 (0.0)	1 (1.4)	
Operation methods (%)			0.530
Laparoscopy	57 (39.6)	23 (31.9)	
Open	75 (52.1)	43 (59.7)	
Conversion	12 (8.3)	6 (8.3)	
Stage (%)			0.092
II	48 (33.3)	16 (25.0)	
III	96 (66.7)	56 (77.8)	
CONUT score	2.6 ± 2.2	3.2 ± 2.3	0.181
LN harvest	27.4 ± 14.0	26.8 ± 15.1	0.781
Tumor size (cm)	5.5 ± 2.1	5.6 ± 2.2	0.791
Lymphatic invasion, Yes (%)	92 (63.9)	44 (61.1)	0.690
Vascular Invasion, Yes (%)	22 (15.3)	13 (18.1)	0.601
Perineural invasion, Yes (%)	52 (36.1)	28 (38.9)	0.690
Differentiation (%)			0.840
Well	9 (6.3)	4 (5.6)	
Moderate	125 (86.8)	63 (87.5)	
Poor	10 (6.9)	5 (6.9)	

*CEA* carcinoembryonic antigen, *ASA* American Society of Anesthesiologist physical status, *LN* lymph node, *CONUT* Controlling Nutritional Status

**Table 2:** Associations between body composition factors and pre-sarcopenia

	Non-pre-sarcopenia			Pre-sarcopenia		
Sex	Male (*N* = 75)	Female (*N* = 69)	*P*	Male (*N* = 51)	Female (*N* = 21)	*P*
BMI (kg/m^2^)	23.9 ± 2.6	23.6 ± 3.2	0.486	21.2 ± 2.7	21.0 ± 3.4	0.687
Visceral fat area (cm^2^)	129.2 ± 69.6	87.2 ± 56.8	< 0.001	101.6 ± 64.0	52.7 ± 39.8	< 0.001
Subcutaneous fat area (cm^2^)	88.9 ± 39.8	136.9 ± 57.9	< 0.001	68.3 ± 39.7	87.2 ± 45.3	0.083
Abdominal circumference (cm)	86.9 ± 8.8	82.9 ± 9.2	0.008	83.4 ± 9.3	74.3 ± 9.6	< 0.001
SMI (cm^2^/m^2^)	52.7 ± 4.2	43.8 ± 4.4	< 0.001	118.2 ± 11.5	83.1 ± 7.0	< 0.001

*BMI* body mass index, *SMI* skeletal muscle index

At a median follow-up of 54 months (range, 2–137), there was no significant difference of short-term outcomes between pre-sarcopenia and non- pre-sarcopenia groups. A comparison of the clinical outcomes between patients with or without pre-sarcopenia is summarized in Table 3. A comparison of the clinical outcomes between patients with or without pre-sarcopenia is summarized in Table 3. There were no significant differences between stent insertion and pre-sarcopenia in obstructive colon cancer patients or in the frequencies of complications such as anastomosis leak, postoperative ileus, and surgical site infection. Patients with pre-sarcopenia showed a significantly shorter median DFS than patients without pre-sarcopenia (median [IQR] 29.5 [11.8–109.0] versus 42.0 [24.0–133.0]; *p* = 0.007). Similarly, overall survival (OS) was shorter in patients with pre-sarcopenia than in patients without pre-sarcopenia (median [IQR] 38.0 [21.8–60.3] versus 58.0 [26.0–81.0] days; *p* = 0.047) (Table 3).

**Table 3:** Post-operative and clinical outcomes according to pre-sarcopenia status

	Non-pre-sarcopenia (*N* = 144)	Pre-sarcopenia (*N* = 72)	*P*
Postoperative hospital days (median [IQR])	13.0 [10.0–17.0]	14.0 [10.8–17.0]	0.450
Stent, yes (%)	80 (55.6)	37 (51.4)	0.562
Stoma, yes (%)	25 (17.4)	16 (22.2)	0.390
Emergency operation, yes (%)	9 (6.3)	6 (8.3)	0.570
Transfusion, yes (%)	66 (45.8)	41 (56.9)	0.124
Complications (%)			0.906
No	119 (82.6)	58 (80.6)	
Minor^a^	17 (11.8)	9 (12.5)	
Major^b^	8 (5.6)	5 (6.9)	
Anastomosis leak (%)	7 (4.9)	4 (5.6)	0.827
Postoperative ileus (%)	8 (5.6)	3 (4.2)	0.662
Surgical site infection (%)	6 (4.2)	2 (2.8)	0.610
Anastomosis site bleeding (%)	2 (1.4)	0 (0.0)	0.315
Urinary retention (%)	2 (1.4)	2 (2.8)	0.475
Atelectasis (%)	4 (2.8)	3 (4.2)	0.587
Pleural effusion	1 (0.7)	1 (1.4)	0.615
Asthma attack	1 (0.7)	0(0.0)	0.478
Recurrence, yes (%)	30 (20.8)	23 (31.9)	0.074
Death, yes (%)	20 (13.9)	21 (29.2)	0.007
Disease-free survival time (months) (median [IQR])	42.0 [24.0–133.0]	29.5 [11.8–109.0]	0.007
Overall-survival time (months) (median [IQR])	58.0 [26.0–81.0]	38.0 [21.8–60.3]	0.047

^a^Clavien-Dindo Classification < IIIb

^b^Clavien-Dindo Classification ≥ IIIb

**Fig. 1:**
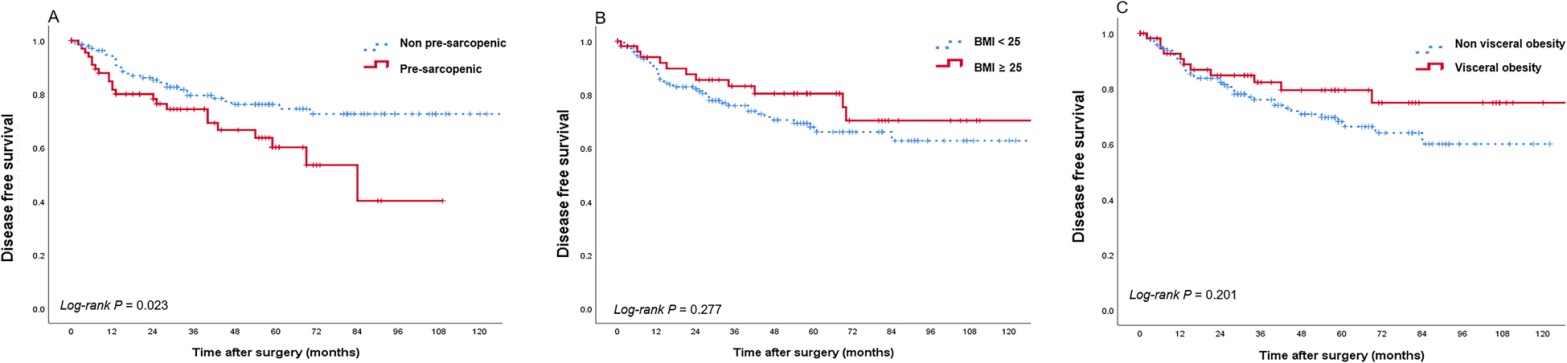
Kaplan–Meier curve of disease-free survival according to body composition and systemic inflammation: skeletal muscle index (**a**), body mass index (**b**), visceral fat (**c**), and neutrophil-lymphocyte ratio (**d**)

During the follow-up period, 53 cancer recurrence events (24.5%) were observed. Patients with pre-sarcopenia showed a significantly shorter DFS than patients without pre-sarcopenia (hazard ratio [HR] = 1.860, 95% confidence interval [CI] 1.08–3.22, *p* = 0.023) (Fig. 1a; Table 4). CONUT score, which represents nutritional state, were not associated with DFS (*p* = 0.892). Visceral adiposity and BMI were not associated with DFS (*p* = 0.206, 0.265, and 0.702, respectively) (Fig. 1b, c).

**Table 4:** Disease-free survival analysis by clinicopathologic variables and body composition factors

Variable	Analysis			
	Univariate		Multivariable	
	HR [95% CI]	*P value*	HR [95% CI]	*P value*
Sex, male	1.09 [0.63–1.88]	0.818	–	–
Age > 60	1.03 [0.60–1.78]	0.908		
ASA (I vs II + III + IV)	1.08 [0.60–1.94]	0.790	–	–
Stage (II vs III)	6.27 [2.13–17.39]	< 0.001	3.64 [1.14–11.67]	0.030
Vascular invasion	2.05 [1.09–3.84]	0.026	1.77 [0.92–3.37]	0.087
Lymphatic invasion	3.81 [1.72–8.44]	0.001	1.76 [0.70–4.43]	0.229
Pre-operative CEA level > 5	2.17 [1.25–3.75]	0.006	2.226 [1.28–3.87]	0.005
Neutrophil lymphocyte ratio ≥ 5	0.31 [0.35–1.34]	0.702		
Differentiation (well vs moderate +poor)	1.70 [0.41–7.01]	0.461	–	–
BMI ≥ 25	0.69 [0.36–1.35]	0.265	–	–
Visceral obesity ≥ 130[33]	0.65 [0.34–1.27]	0.206	–	–
Subcutaneous fat area (cm^2^) > 92^a^	0.80 [0.47–1.37]	0.417	–	–
Abdominal circumference (cm) > 83^a^	0.89 [0.52–1.52]	0.663	–	–
CONUT score > 7[22]	0.91 [0.22–3.79]	0.892	–	–
Pre-sarcopenia, men < 46.4 cm^2^, women < 37.5 cm^2^ [32]	1.83 [1.08–3.22]	0.023	1.86 [1.04-3.13]	0.037

*CI* confidence interval, *ASA* American Society of Anesthesiologist physical status, *CEA* carcinoembryonic antigen, *BMI* body mass index, *CONUT* Controlling Nutritional Status

^a^Median value

**Fig. 2:**
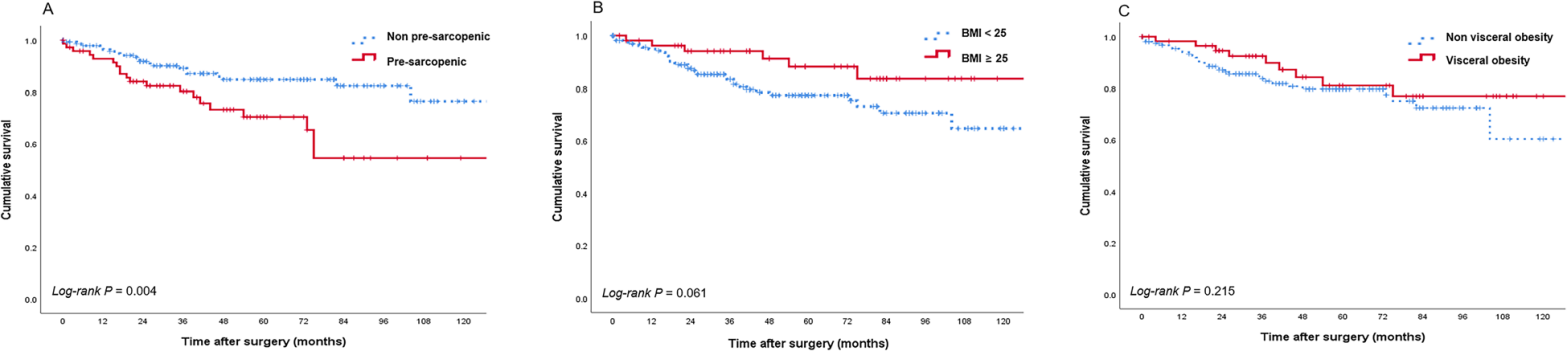
Kaplan–Meier curve of overall survival according to body composition and systemic inflammation: skeletal muscle index (**a**), body mass index (**b**), visceral fat (**c**), neutrophil–lymphocyte ratio (**d**)

On univariate analyses, Age, ASA, stage, vascular invasion, CONUT score, and pre-sarcopenia were associated with OS. Pre-sarcopenia patients showed a significantly shorter OS than non-pre-sarcopenia patients (HR = 2.38, CI 1.29–4.41, *p* = 0.004) (Fig. 2a). In multivariable adjusted analysis, pre-sarcopenia (HR = 1.92 CI 1.02 to 3.60, *p* = 0.043) was retained as a risk factor of OS for a 5-year OS (Table 5). Visceral adiposity and BMI were not associated with OS (*p* = 0.061, 0.215, and 0.527, respectively; Fig. 2b, c).

**Table 5:** Overall survival analysis by clinicopathologic variables and body composition factors

Variable	Analysis			
	Univariate		Multivariable	
	HR [95% CI]	*P value*	HR [95% CI]	*P value*
Sex, male	1.43 [0.75–2.74]	0.284	–	–
Age > 60	2.88 [1.37–6.04]	0.005	2.51 [1.14–5.56]	0.023
ASA (I vs II + III + IV)	2.27 [1.01–5.14]	0.048	1.99 [0.84–4.71]	0.118
Stage (II vs III)	2.78 [1.17–6.64]	0.021	2.29 [0.94–5.59]	0.069
Vascular invasion	2.14 [1.04–4.40]	0.039	1.90 [1.01–3.57]	0.030
Lymphatic invasion	1.97 [0.94–4.13]	0.074	–	–
Pre-operative CEA level > 5	1.63 [0.84–3.13]	0.147	–	–
Neutrophil lymphocyte ratio ≥ 5	0.78 [0.36–1.70]	0.529		
Differentiation (well vs moderate +poor)	0.66 [0.21–1.67]	0.322	–	–
BMI ≥ 25	0.45 [0.19–1.06]	0.069	–	–
Visceral obesity ≥ 130[33]	0.63 [0.22–1.32]	0.220	–	–
Subcutaneous fat area (cm^2^) > 92^a^	0.81 [0.43–1.51]	0.504	–	–
Abdominal circumference (cm) > 83^a^	0.81 [0.44–1.52]	0.817	–	–
CONUT score > 7[22]	2.27 [0.53–9.61]	0.254		
Pre-sarcopenia, men < 46.4 cm^2^, women < 37.5 cm 2[32]	2.38 [1.29–4.41]	0.004	1.92 [1.01–3.57]	0.047

*CI* confidence interval, *ASA* American Society of Anesthesiologist physical status, *BMI* body mass index, *CEA* carcinoembryonic antigen, *CONUT* Controlling Nutritional Status

^a^Median value

## Discussion

Pre-sarcopenia was negatively associated with a long-term oncological prognosis. Pre-sarcopenia patients showed significantly lower OS and DFS. However, there were no significant differences in short-term postoperative outcomes. Body composition factors other than pre-sarcopenia did not significantly impact the patient’s survival. Pre-sarcopenia was negatively associated with a long-term oncological prognosis. Pre-sarcopenia patients showed significantly lower OS and DFS. However, there were no significant differences in short-term postoperative outcomes. Body composition factors other than pre-sarcopenia did not significantly impact the patient’s survival.

Pre-sarcopenia comprises both muscle loss and its dysfunction, which induce contractile impairment and metabolic and endocrine abnormalities. It affects whole-body metabolism and the immune or inflammatory response [35, 36]. The loss of muscle and the accumulation of intramuscular fat might be associated with metabolic syndrome via a complex interplay of factors including oxidative stress, proinflammatory cytokines, insulin resistance, hormonal changes, and mitochondrial dysfunction [37]. These factors may have contributed to long-term survival.

The results of our study suggest that pre-sarcopenia is a negative prognostic factor for both OS and DFS in patients with obstructive colorectal cancer. To our knowledge, this is the first report to describe the prognostic impact of pre-sarcopenia specifically in patients with obstructive colorectal cancer. Other studies reported pre-sarcopenia to have a negative effect on OS in patients undergoing resection for locally advanced rectal cancer after neoadjuvant chemoradiation therapy [15] and as an independent predictor of worse OS and DFS in stage I to III colorectal cancer [38].

Early studies of pre-sarcopenia were often based on the work by Prado et al. [39]. In their study, they included pre-sarcopenia data based on solid tumors of the lung or gastrointestinal tract from patients referred to a regional medical oncology service in Canada. However, because there are significant differences in body composition between different ethnicities, more data are needed from the Asian populations [40]. Moreover, more data are needed to assess the optimal cutoff value for pre-sarcopenia for each ethnicity [13]. The Asian cut-off value for pre-sarcopenia should be different from the one used in Western countries.

Pre-sarcopenia was associated with a significantly increased risk of developing major complications [41]. However, one study reported that pre-sarcopenia was not a predictor of postoperative complications [8]. In the present study, no significant differences in minor (11.8% vs. 12.5%) or major postoperative complications (5.6% vs. 6.9%) were observed between non-pre-sarcopenia and pre-sarcopenia patients (Table 3). All surgeries were performed at the three tertiary-referral hospitals where more than 100 colorectal cancer patients are treated annually by seven independent surgeons. These surgeons were qualified through live demonstrations held by the Korean Laparoscopic Colorectal Surgery Study Group, and each submitted a videotape of their laparoscopic rectal surgery, which was subsequently reviewed by a trial steering committee to assess the surgeon’s oncological technique [42].

Visceral obesity was reported to be a significant prognostic factor in predicting DFS in patients with resectable colorectal cancer [43]. Similarly, viscerally obese patients with rectal cancer have poorer DFS [44]. In contrast, BMI measurements were not correlated with any survival outcomes [43, 44]. Our data demonstrated that pre-sarcopenia was negative associated with visceral obesity (Table 2), but there were no other significant differences in predicting DFS and OS in patients with obstructive colorectal cancer. A lower BMI was correlated with pre-sarcopenia but not with prognosis. SMI was the most meaningful prognostic value among body composition factors.

According to a recent meta-analysis study [13], most studies (11/22, 50%) used SMI when applying muscle mass criteria in CT scan-based assessments of skeletal muscle index (SMI). Their defined cutoff values varied between 53.5 and 40.8 cm^2^/m^2^ for men and 46.4 and 34.9 cm^2^/m^2^ for women. To define a pre-sarcopenia cutoff value for this study, we analyzed the association between body composition factors and pre-sarcopenia in four different studies that included an Asian study population (46.4, 43.75, 43.2, and 40.8 cm^2^/m^2^ for men and 37.5, 41.10, 35.3, and 34.9 cm^2^/m^2^ for women for each study, respectively) [32, 45–47]. Among these, the cutoff value proposed by Takagi was applied differently to men and women and the inclusion criteria selected in their study was similar to ours. Additionally, their selected cutoff value did not differ significantly from the cutoff value of other Asian references. Thus, we used the same cutoff value proposed by Takagi [32], which was ideal for our study in Asian patients.

There are some limitations to this study. One possible limitation of the study is the definition of pre-sarcopenia. It is based on the definition by the European Working Group on Sarcopenia in Older People (EWGSOP) and the Asian Working Group for Sarcopenia (AWGS) that relies on the presence of low muscle mass and low muscle function (muscle strength and physical performance) [11]. However, there is still no uniform standard to measure and define sarcopenia, including the protocol of grip strength measures till date [48, 49]. Pre-sarcopenia can be measured by CT scan by assessing the muscle area in the L3 region [10]. This method is commonly used both to measure SMI and to define pre-sarcopenia.

Another limitation of the study is bias of SMI results for the measurement of pre-sarcopenia. There were no association between pre-sarcopenia and nutrition status in this study (Supplementary Table 1&2). That is, clinicians were not blinded to the SMI results. Since this is a retrospective study, surgeons may have been more careful to perioperative management such as nutrition support to those with pre-sarcopenia during and after surgery. It is apparent that patients who had pre-sarcopenia were well managed in this study, as reflected by the complication rate with no significant difference shown between pre-sarcopenia and non-pre-sarcopenia patients. Owing to these limitations, the conclusion of our study may not be definite, and hence, the differences in postoperative outcome between pre-sarcopenia and long-term outcomes in obstructive colon cancer must be probed further with prospective randomized studies. Despite these limitations, the results of our study emphasized that the pre-sarcopenia diagnosed by L3SMI showed oncologic significance in obstructive colorectal cancer.

Treatment of obstructive colorectal cancer may occasionally require colonic stent implantation to improve the patient’s condition along with adjuvant chemotherapy that is often administered to these patients. Patients with obstructive colon cancer generally have poor prognosis and suffer large clinical burdens, including pre-sarcopenia.

The CONUT score was originally developed as a tool for nutritional assessment and is reported to be significantly associated with the prognosis of colorectal cancer [50]. Additionally, the preoperative CONUT score was an independent prognostic factor for cancer specific and disease-free survival in obstructive colorectal cancer patients [22]. Results of this study suggest that pre-sarcopenia is still considered to be an important factor in obstructive colorectal cancer, regardless of the patient’s nutritional status or inflammatory condition.

In conclusion, a careful consideration and analysis of the patient’s body composition status should be performed to overcome the large clinical burden in patients with obstructive colon cancer. In particular, pre-sarcopenia should be considered in the patients’ risk assessments and stratification of oncologic prognosis. The pre-sarcopenia, diagnosed by L3SMI, may be beneficial as a convenient, objective, and noninvasive marker, to guide individualized treatment decisions and improve follow-up outcomes in patients with colorectal cancer.

## Data Availability

The study data is not available

## Abbreviations

CT: Computed tomography
BMI: Body mass index
NLR: Neutrophil to lymphocyte ratio
DFS: Disease-free survival
OS: Overall survival
SMI: Skeletal muscle index
SEMS: Self-expandable metallic stent
